# Adynamic bone disease

**DOI:** 10.1590/2175-8239-JBN-2021-S108

**Published:** 2021-12-03

**Authors:** Ana Paula Santana Gueiros, Rodrigo Azevedo de Oliveira, Aluizio Barbosa Carvalho

**Affiliations:** 1 Hospital das Clínicas da Universidade Federal de Pernambuco, Recife, PE, Brazil.; 2 Universidade Federal do Rio Grande do Norte, Department of Integrated Medicine, Natal, RN, Brazil.; 3 Universidade Federal de São Paulo, Department of Medicine, Nephrology Division, São Paulo, SP, Brazil.

## 1. Diagnosis of adynamic bone disease

1.1. The presence of adynamic bone disease (ABD) should be suspected in elderly, diabetic, parathyroidectomized patients intensively treated with calcimimetics, calcitriol or analogues, and in those exposed to aluminum or calcium overload either orally or by dialysate with high calcium concentration (3.5mEq/L) for long term (Evidence).

1.2. Bone biopsy is the gold standard method for the diagnosis of ABD (Evidence).

1.3. In dialysis patients, serum iPTH levels lower than 2 times the upper limit of the method, especially if associated with normal/reduced alkaline phosphatase (AP) levels, are highly suggestive of ABD (Evidence).

1.4. Increased serum levels of total AP in patients without liver disease, or elevation of bone specific AP, practically exclude ABD (Evidence).

1.5. Bone biopsy or desferrioxamine test should be performed in case of aluminum-associated ABD suspection (Evidence).

## 2. Treatment of adynamic bone disease

2.1. Factors that contribute for increased bone resistance to PTH such as hyperphosphatemia, malnutrition, corticoid use, hypogonadism, among others, should be avoided (Opinion). 

2.2. Therapies that contribute to the suppression of serum iPTH levels, as calcium-based phosphate binders, calcitriol or its analogues, calcimimetics, and dialysate with a calcium concentration of 3.5 mEq/L, should be avoided (Evidence).

2.3. Calcium-free phosphate binders, such as sevelamer hydrochloride, should be preferably used for controlling serum phosphorus levels (Evidence).

2.4. Desferrioxamine is the drug of choice for ABD treatment associated with aluminum toxicity (Evidence).

## Rational

Adynamic bone disease (ABD) represents a well-defined clinical entity among chronic kidney disease-mineral and bone disorders (CKD-MBD).^1^ ^-^ ^3^ Its prevalence has been increasing over the last three decades, reaching up to 50% and 70% of patients with CKD G2-5 and 5D, respectively.^3^ ^-^ ^8^ This increase in the prevalence of ABD is due, among other factors, to the greater number of elderly and diabetic patients with CKD and to more intensive treatment of secondary hyperparathyroidism.^3^

ABD is characterized by low bone turnover, with decreased bone formation rate and, consequently, poor osteoid matrix. Cellularity is scarce (osteoblasts and osteoclasts) and there is no bone marrow fibrosis.

It is a disease with few symptoms, except when associated with aluminum toxicity, a situation that usually causes bone pain and muscle weakness. However, it is associated with a higher risk of fractures and vascular calcification, corroborating unfavorable outcomes.^2^ ^,^ ^6^ ^,^ ^9^ ^,^ ^10^

Two conditions are determinant in the pathogenesis of ABD: suppression of parathormone (PTH) secretion and skeletal resistance to the action of this hormone.^2^ Suppression of PTH secretion or relative hypoparathyroidism state is usually a consequence of excessive use of calcitriol or analogues, and calcimimetics, as well as calcium overload. The use of calcium-based phosphate binders and the high concentration of calcium in the dialysate are important contributors to that overload. Other factors may also contribute to lower PTH levels and low bone turnover, such as advanced age, *diabetes mellitus*, peritoneal dialysis, hypogonadism, malnutrition, corticosteroid therapy, bisphosphonate use, aluminum toxicity, and parathyroidectomy (PTX).^2^ ^,^ ^11^ ^-^ ^13^

Currently, aluminum toxicity is still a reality. The Brazilian Registry of Bone Biopsy (Rebrabo) detected the presence of aluminum in the bone trabeculae of 38% of biopsies from CKD patients.^14^ Some previous publications state that aluminum bone toxicity is very rare in the contemporary world.^15^ ^-^ ^17^ However, these are old studies and some of them are based only on serum aluminum levels. We believe that such controversy is due to regional characteristics, but mainly to underdiagnosis.

A number of factors are involved in bone resistance to PTH action, such as phosphorus overload, calcitriol deficiency, decreased expression of PTH receptors in bone tissue, and the presence of uremic toxins.^7^ ^,^ ^13^ Sclerostin and Dickkopf-related protein 1 (DKK1) are bone formation inhibitors, since they negatively regulate the osteoblast maturation pathway. For this reason, they may develop an important role in the pathogenesis of ABD and have therapeutic implications. The anabolic effect of PTH on bone seems to be mediated by suppression of sclerostin activity.^18^ ^-^ ^20^ There is an important association between the presence of *diabetes mellitus* and higher levels of sclerostin - both serum and expressed in bone tissue.^21^

Bone biopsy is the gold standard for diagnosing ABD, although its invasive nature and lack of availability in most centers are factors that prevent it from being routinely performed. Thus, in daily practice, the diagnosis of ABD is based on the use of biochemical and hormonal parameters that reflect bone turnover, especially intact PTH (iPTH) and total AP or, preferably, its bone fraction.^1^ ^,^ ^22^ Serum iPTH levels lower than 150 pg/mL (2 times the upper limit for the method) are good predictors of ABD.^22^ This predictive value is higher when associated with low levels of total or bone AP. Elevated bone specific AP (≥ 20 ng/mL), either isolated or associated with iPTH level greater than 200 pg/mL, is able to exclude ABD.^23^ Patients with iPTH levels within the KDIGO recommended range (2-9 times the normal threshold) may have ABD.^1^

ABD patients have abnormal calcium homeostasis, characterized by difficulty in incorporating this mineral into the bone. This implies a greater risk of hypercalcemia in case of calcium overload.^24^ Regardless of its origin, oral or dialysate, calcium overload leads to a vicious cycle of PTH suppression and low bone calcium incorporation, favoring the development of extraosseous calcification. Thus, the use of calcium-based phosphate binders as well as dialysate with calcium concentrations higher than 3.0 mEq/L is not recommended for patients with ABD^1^ ^,^ ^25^. The use of low calcium dialysate is an auxiliary tool to increase PTH secretion, which has been associated with restoration of bone turnover.^26^ ^-^ ^29^

Another possible way to improve bone turnover would be the use recombinant PTH, such as teriparatide (PTH 1-34), which is capable of promoting osteoblastic and osteoclastic activity.^30^ ^,^ ^31^ However, few studies have evaluated the use of teriparatide in patients with CKD-MBD. This drug has already been used in a few patients with ABD, resulting in increased bone turnover, volume, and mineral density, besides controlling the ABD-associated hypercalcemia.^32^ ^-^ ^40^ Clinical trials are needed to demonstrate the safety and efficacy of teriparatide in ABD treatment.

To date, there are no major prospective, randomized, controlled studies that firmly support the ABD treatment. In summary, its current therapy follows two basic principles: reducing calcium exposure and raising PTH levels. A thorough review of the medical prescription should be encouraged, aiming at the suspension of calcium salts, calcimimetics, calcitriol and its analogues. Reducing calcium concentration of the dialysate is another important measure.
